# Discrepancy in coordination and variation of root and leaf traits among herbaceous and shrub species in the desert, China

**DOI:** 10.3389/fpls.2024.1485542

**Published:** 2024-11-12

**Authors:** Jing Ma, Taotao Wang, Hongyong Wang, Yiming Chen, Jie Yang, Tingting Xie, Lishan Shan

**Affiliations:** ^1^ College of Forestry, Gansu Agricultural University, Lanzhou, China; ^2^ State Key Laboratory of Desert and Oasis Ecology, Xinjiang Institute of Ecology and Geography, Chinese Academy of Sciences, Urumqi, China; ^3^ Xinjiang Key Laboratory of Desert Plant Roots Ecology and Vegetation Restoration, Xinjiang Institute of Ecology and Geography, Chinese Academy of Sciences, Urumqi, China; ^4^ Cele National Station of Observation and Research for Desert-Grassland Ecosystems, Hotan City, Cele, Xinjiang Uygur Autonomous Region, China

**Keywords:** life form, hub traits, leaf economics spectrum, root economics spectrum, ecological strategy

## Abstract

**Introduction:**

Alterations in life forms could simultaneously influence the variability of fine root and leaf traits. However, our understanding of the organ-level coordination and patterns of variation in fine root and leaf traits among desert herbs and shrubs with distinct habits remains limited.

**Methods:**

Consequently, this study examined the leaf and fine root traits of 9 shrubs and 9 herbs across three desert habitats through Sperman's correlation analysis, principal component analysis (PCA), and standardized major axis (SMA) analysis as a means of identifying the hub traits and the resource trade-off strategies employed by desert plants to adapt to their environment.

**Results:**

The results showed that the extent of coordination between leaf and root traits, defined as similarity, was contingent upon the life form. While the traits in shrubs were completely decoupled, those in herbs exhibited a high degree of coordination. The traits related to water acquisition and storage are highly connected and do not depend on traits and life forms. Most leaves and fine roots were primarily loaded along the PC1 and PC2 axes of principal component analysis.

**Discussion:**

Suggesting that herbs and shrubs each adopt the most advantageous trait syndrome in accordance with their life form to acquire and conserve resources. The leaf economic spectrum and the root economic spectrum evolved independently, showing no dependence on the variations in life form. In conclusion, in desert plants, leaf and root variations occur independently along two axes, with traits associated with water acquisition playing a neutral role in their ecological strategy.

## Introduction

Plant is a component organism composed of three main components including root, stem and leaf, which are systematically coordinated (Chave et al., 2009). Leaf is the main organ that can capture light and fix CO_2_ for photosynthesis (Freschet et al., 2010). Stems can support leaves and transport water and nutrients between roots and leaves. Many plants also store a large amount of water and nutrients in their stems (Reich, 2014; Li et al., 2022). The roots anchor the whole plant and acquire water and available nutrient elements from the soil, and temporarily store them (Freschet et al., 2021; Han and Zhu, 2021). As a consequence, leaves and fine roots are the main organs to capture resources (Cheng et al., 2016; Zhou et al., 2024) and stems are nutrient transport organs (Li et al., 2022), which together affect the overall adaptability of an individual plant to the environment. The degree of interdependence between different organ traits of plants determines the coordination and response of different plant components to environmental changes (Stearns, 1989; Freschet et al., 2015b). Plant functional traits defined as hub traits usually have a high degree of connection with other traits and are located between multiple sub-networks (Kleyer et al., 2019). Variations of a hub trait can affect several sub-traits that play an important role in trait trade-offs (Cohen and Havlin, 2010; Messier et al., 2017).

Based on the variation and trade-off of leaf functional traits globally, Wright et al. (2004) defined a continuously changing one-dimensional leaf economics spectrum (LES) to explain the trade-off between leaf resource acquisition and preservation (Wright et al., 2005; Haynes, 2022). One end of the LES represents the quick-return on investment strategy, i.e., these species typically have larger specific leaf area, leaf nitrogen content, and faster photosynthetic rates as a means of achieving rapid access to light energy and carbon resources, but lower leaf thickness and tissue density and shorter lifespans. Species with this strategy have lower tissue investment and faster return on investment (acquisition strategy). The other end of LES indicates the slow-return on investment strategy, i.e., these species typically have higher tissue densities and leaf thicknesses, longer lifespans to retain resources, but lower specific leaf area, leaf nitrogen content, and photosynthetic rates. Species with this feature have higher tissue investment and lower rates of return (conservative strategy) (Reich, 2014; Fajardo and Siefert, 2018). Therefore, by studying LES, we can better understand the resource allocation strategies of plants at the leaf level and the external manifestations of this physiological function. This contributes to our understanding of ecosystem material cycling and energy flow, as well as explaining the mechanisms of species coexistence in plant communities and predicting the response of plant communities to environmental changes (e.g., climate, land use, etc.). At present, LES has been found on many scales, including interspecies, intraspecies, and even within individual plants(i.e. different parts of the individual plant) (Vasseur et al., 2012; Blonder et al., 2015; Zhou et al., 2024). With further research, many scholars have extended the resource economy spectrum to stem(Sakschewski et al., 2015; Zeballos et al., 2017), root (Isaac et al., 2017; Hogan et al., 2020; Carmona et al., 2021; Yu et al., 2022), whole-plant (Stahl et al., 2013; Reich, 2014; Li et al., 2022; Zhang et al., 2023), and community levels (Carvajal et al., 2019).

The traditional root economics spectrum (RES) represents a trade-off strategy between resource acquisition efficiency and resistance (Prieto et al., 2015). One side of the RES is a coarse-root plant with a conservative strategy, and the other side is a fine-root plant with an acquisition strategy (Ma et al., 2018). However, more and more evidences show that the variation of root traits is two-dimensional (Kramer-Walter et al., 2016; Weemstra et al., 2016; Ding et al., 2020; Carmona et al., 2021). Specifically, in addition to the fast-slow axis, root traits also cover the second dimension of the collaboration axis (Zhang et al., 2024). Fine root species with high specific root length (SRL, root length per unit root dry weight) are separated from coarse root species that are more dependent on mycorrhizal symbionts for nutrient capture (Bergmann et al., 2020). This ecological strategy means that traits that are similar in definition, such as specific leaf area (SLA) and specific root length (SRL), are functionally different. Although leaves with lower SLA are slower in aboveground resource capture, roots with low SLA can still achieve higher resource acquisition efficiency based on higher mycorrhizal fungal colonization rates (Kong et al., 2014; Cheng et al., 2016).

Ecological research based on the resource economics spectrum can help us understand the resource-economy trade-offs in different organs (Zhang et al., 2023). It is worth noting that the variation and coordination of above-ground and below-ground functional traits are limited to some extent by environmental conditions due to the general trade-off of resource allocation within plants (Hu et al., 2019; Ma et al., 2022; Luo et al., 2024). Although some of the existing research results provide a theoretical basis for us to understand the variation patterns of plant organ traits between and within species, and their adaptation strategies to environmental conditions (Zhang et al., 2023; Li et al., 2024; Zhou et al., 2024). However, there is very little data on whether leaf and root functional traits have similar variation patterns (Valverde-Barrantes et al., 2015), or whether they respond to environmental gradients in a coordinated manner among species (Wang et al., 2017).

Based on this situation, life forms may be a useful entry point for identifying common patterns of variation and coordination among species (Liu et al., 2019). Plants from different life forms are more affected by evolution and environment (Ma et al., 2024). Specifically, plant size, woodiness, life span, and root-leaf turnover rate, vary greatly among different growth forms (Dorrepaal, 2007). Similarly, the differences in life history strategies between woody and herbaceous species lead to differences in resource acquisition and conservation strategies (Freschet et al., 2017; Kong et al., 2019). For instance, some studies have found that herbaceous species with roots with higher SRL and root nitrogen content(RNC) could simultaneously have higher SLA and Leaf nitrogen content(LNC) (Craine et al., 2002; Tjoelker et al., 2005). However, the traits in woody plants are just the opposite (Tjoelker et al., 2005; Weemstra et al., 2016). This indicates that growth forms may simultaneously regulate the variation of fine root and leaf traits, but little is known about the coordination and variation patterns of fine root and leaf traits in desert herbaceous and shrubs species.

Herbaceous and Shrubs plants play an important role in plant composition of terrestrial ecosystems, especially desert ecosystems, which account for more than 35% of the world’s land area (Mares, 1999). Desert plants have developed specific morphological and physiological traits to adapt to arid environment through long-term evolutionary selection. Generally, plants can mitigate the water limitation on their growth and development in arid environments by reducing leaf area, increasing leaf thickness and relative water content, and increasing root surface area and volume. With regard to the relationship between leaves and roots, a large number of studies have concluded that their functional properties are highly compatible (Freschet et al., 2010; De La Riva et al., 2016), however, Isaac et al., 2017 found that root traits vary with environmental conditions, but not necessarily with leaf traits and their function. Given this, this study takes 9 shrubs and 9 herbs in the arid desert area of Northwest China as the research object, and combines their differences in life history strategies and the characteristics of arid and sparse vegetation in a desert environment, we hypothesized that (i) Hub traits varied with different life forms, but generally the traits related to nutrients acquisition and preservation were more neutral, and (ii) the resource strategies are different due to different life forms that shrubs are more dependent on leaf variation to adapt to the environment, while herbs are just the opposite, and (iii) the decoupling of leaf and root trait variation and the independent adaptation to the environment for both shrubs and herbs, which mean the risk sharing in harsh environments.

## 2 Materials and methods

### 2.1 Study sites

**Figure 1:**
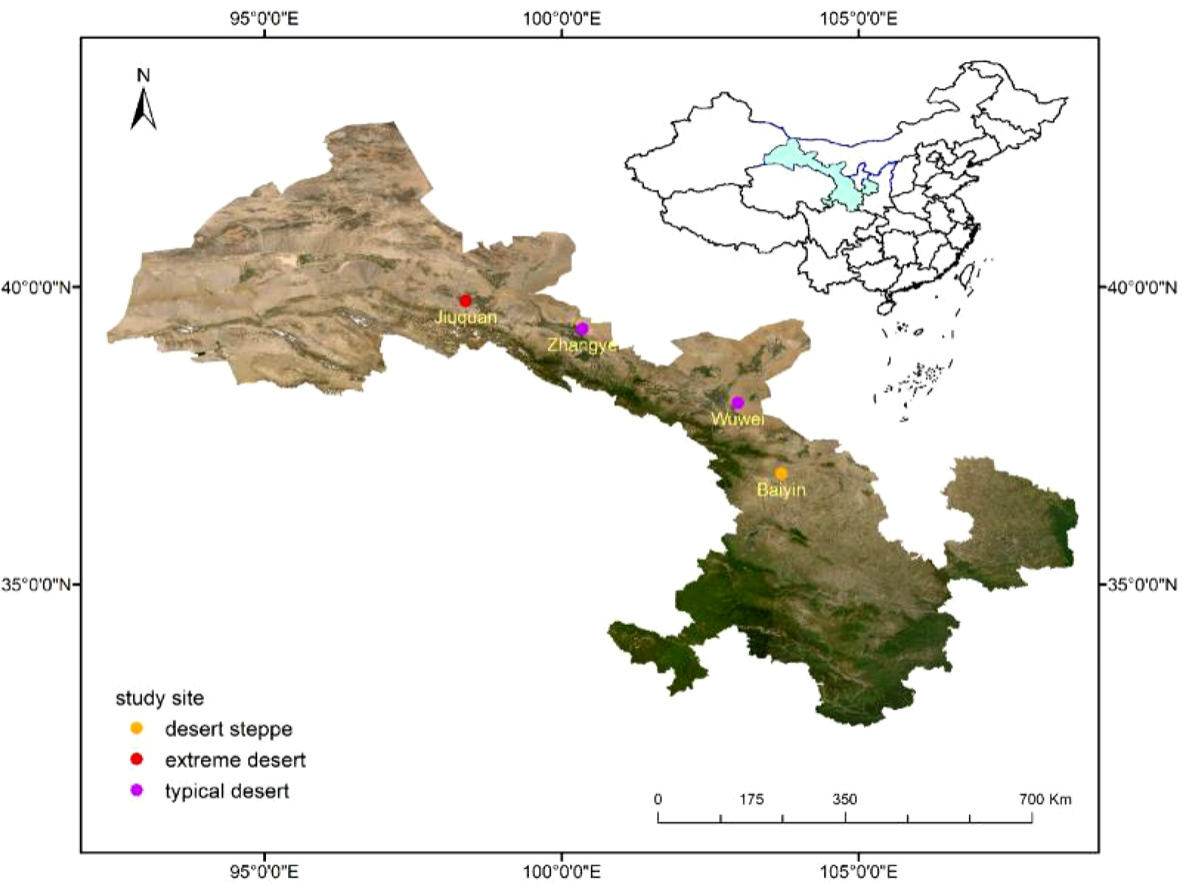
This study established four sampling sites across different desert and grassland ecosystems, which include the desert grassland of Baiyin, the typical deserts of Zhangye and Wuwei, and the extreme desert conditions found in Jiuquan.

Field sampling was conducted in the typical desert region of Northwest China. Four desert plant communities, widely distributed along the precipitation gradient from west to east, were chosen as experimental sites. These sites represent extreme desert, typical desert, and desert steppe ecosystems, respectively (**Figure 1**). The climatic conditions of each station are detailed in **Table 1**.

**Table 1:** General situation of the study area.

Site	JiuQuan	ZhangYe	WuWei	BaiYin
Latitude	39°46′	39°18′	37°63′	36°52′
Longitude	98°23′	100°21′	102°58′	103°42′
Altitude (m)	1500	1590	1775	1947
Annual average temperature(°C)	7.5	6.0	7.8	9.0
Annual average precipitation (mm)	86	131	165	225
Annual average evaporation (mm)	2038	2003	2205	1550

### 2.2 Species selection and samples collection

In July 2022, an investigation was conducted to assess the status of desert plant species and communities at four experimental sites. Specifically, shrub-grass vegetation areas with flat terrain and unaffected by grazing were chosen at each site. Subsequently, three large quadrats (50 m × 50 m) were established at each investigated site, resulting in a total of 12 quadrats across the four study sites. Within each large quadrat, five small quadrats (10m × 10m) were positioned at both ends and midpoints of the diagonal for investigating community structure. The species abundance of vegetation in each quadrat was meticulously recorded. Following the methodology described by Cheng et al. (2016) and considering the species distribution at the investigation site, we selected 18 common species (each with a relative abundance greater than 1%), comprising 9 shrubs and 9 herbs. (**Supplementary Figure S1**).

Based on the survey results, 10 individuals of common species were selected in each large quadrat. Following, 10 fully exposed mature leaves were randomly collected from each individual, and each of the 10 mature leaves from each plant consisted of 3-4 leaves from each of its upper, middle, and lower parts. The collected samples are immediately placed in an ice-plastic bag and transported to the laboratory (Yu et al., 2022). Afterwards, to ensure that the fine roots came from the same sample, we used the ‘ main root tracking method ‘ to excavate the fine root samples of the same plant. Specifically, considering the horizontal and vertical distribution of plant roots, in order to maximize the extraction of fine roots from each plant, we dug out soil cores measuring 1 m × 1 m × 0.4 m in length, width, and depth for herbs, and 1 m × 1 m × 0.8 m for shrubs, that over 75% of shrub roots could be found in this area (Jackson et al., 1996). After that, first of all, we must find our main root, and dig down based on the extension direction of the main root to find the fine roots connected to it. We then carefully removed the surrounding sediment near the end of the root to avoid damage to the fine roots. When the fine roots on a main root are completely exposed or a large number of fine roots appear, the tracking ends. We collected fine root samples (< 2mm) from the 1 ~ 2 roots at the tracking end. The soil and impurities on the root surface were carefully clarified and placed in a labelled self-sealing bag, and then placed in a refrigerator at about-4°C. The root traits were measured in the laboratory.

### 2.3 Plant trait measurements

After the sample was transported back to the laboratory, the fresh weight of each plant leaf was immediately weighed and recorded, and then the Epson Perfection V850 Pro Scanner (Epson, Los Alamitos, CA USA) was used to scan the leaves to obtain a leaf image with a resolution of 600 dpi. After that, the leaves were completely immersed in deionized water for 12 hours in a dark environment. After taking out, the water on the surface of the leaves was quickly absorbed by the absorbent paper and the saturated fresh weight was weighed. After weighing, the leaves transferred into the envelope bag were dried at 75°C for 48 h to constant weight, and the dry weight was weighed and the data were recorded. The degree of leaf succulence (DOF) was calculated as the ratio of leaf fresh weight to dry weight, and the leaf water content (WC) was calculated as the ratio of the difference between leaf fresh weight and leaf dry weight to leaf fresh weight. Leaves were analyzed in Win-RHIZO 2008 (Regent, Instruments Inc., Canada) to obtain leaf area and leaf volume. Specific leaf area (SLA) was calculated as the ratio of leaf area to leaf dry weight, and leaf tissue density (LTD) was calculated as the ratio of leaf dry weight to leaf volume.

Likewise, we carefully cleaned with deionized water to remove impurities from the surface of the roots. The cleaned roots were scanned into 600 dpi images, and then analyzed in Win-RHIZO 2008 (Regent, Instruments Inc., Canada) to obtain root diameter(RD), root length, root surface area and root volume. After the scanning was completed, the roots transferred into the envelope bag were dried at 75°C for 48 h to constant weight, and the dry weight was weighed and the data were recorded. The specific root length (SRL) was calculated as the ratio of root length to root dry weight, the specific root surface area (SRA) was calculated as the ratio of root surface area to root dry weight, and the root tissue density(RTD) was calculated as the ratio of root volume to root dry weight.

The above-mentioned plant samples were dried, crushed, and sieved, and their leaf carbon concentration (LCC), leaf phosphorus concentration (LPC), leaf nitrogen concentration (LNC), root carbon concentration (RCC), root nitrogen concentration (RNC), and root phosphorus concentration (RPC) were measured. Among them, the carbon concentration was determined by potassium dichromate oxidation heating method (Bao, 2000). Total nitrogen concentration was measured by Kjeldahl method (Bao, 2000). The total phosphorus content was determined by vanadium molybdenum yellow colorimetric method (Bao, 2000). and then their stoichiometric ratios (carbon–nitrogen, carbon–phosphorus, and nitrogen–phosphorus) were calculated.

### 2.4 Data analysis

For each species, we took the average of each trait(SLA, LTD, DOF, WC, LCC, LNC, LPC, LCN, LCP and LNP of leaf, and RD, SRL, SRA, RTD, RCC, RNC, RPC, RCN, RCP and RNP of root). The Sperman’s correlation analysis was used to test the relationship between the traits of each organ. The OmicStudio tools calculated correlation coefficient, p value and relation value (https://www.omicstudio.cn/tool/62) and summarized them in Excel. After that, the visualization of the network diagram was realized by using the summarized data in Gephi-0.9.2 software. The coefficient of variation (*CV*) of each trait was calculated as the standard error divided by the mean and then multiplied by 100%.

Principal components analysis (PCA) was used to detect the resource-economic spectrum coordination model at the organ level and the whole-plant level. PC1, the principal component with the largest eigenvalue, represents the direction of greatest variation in the data. PC2, ensuring orthogonality with PC1, has the next largest eigenvalue and captures the second most significant variation. In principal component analysis, PC1 and PC2 simplify data dimensionality by projecting raw data into a new space defined by these components, maximizing information retention while reducing dimensionality. Based on the axis scores of PCA analysis results, the differences in the scores of each axis in different climatic regions were compared by one-way analysis of variance to determine the differences in the root economic spectrum of herb and shrub species resources in different climatic regions. The standardized major axis (SMA) regression method was used to determine the bivariate correlation between the axis scores of PC1 and PC2 in leaves, stems, and roots to determine the differences in slope and intercept between different life-form species (Niklas, 1994; Warton et al., 2006; Vasseur et al., 2012). All data analyses were performed in R 4.3.0.

## 3 Results

### 3.1 Correlation of root and leaf traits

**Figure 2:**
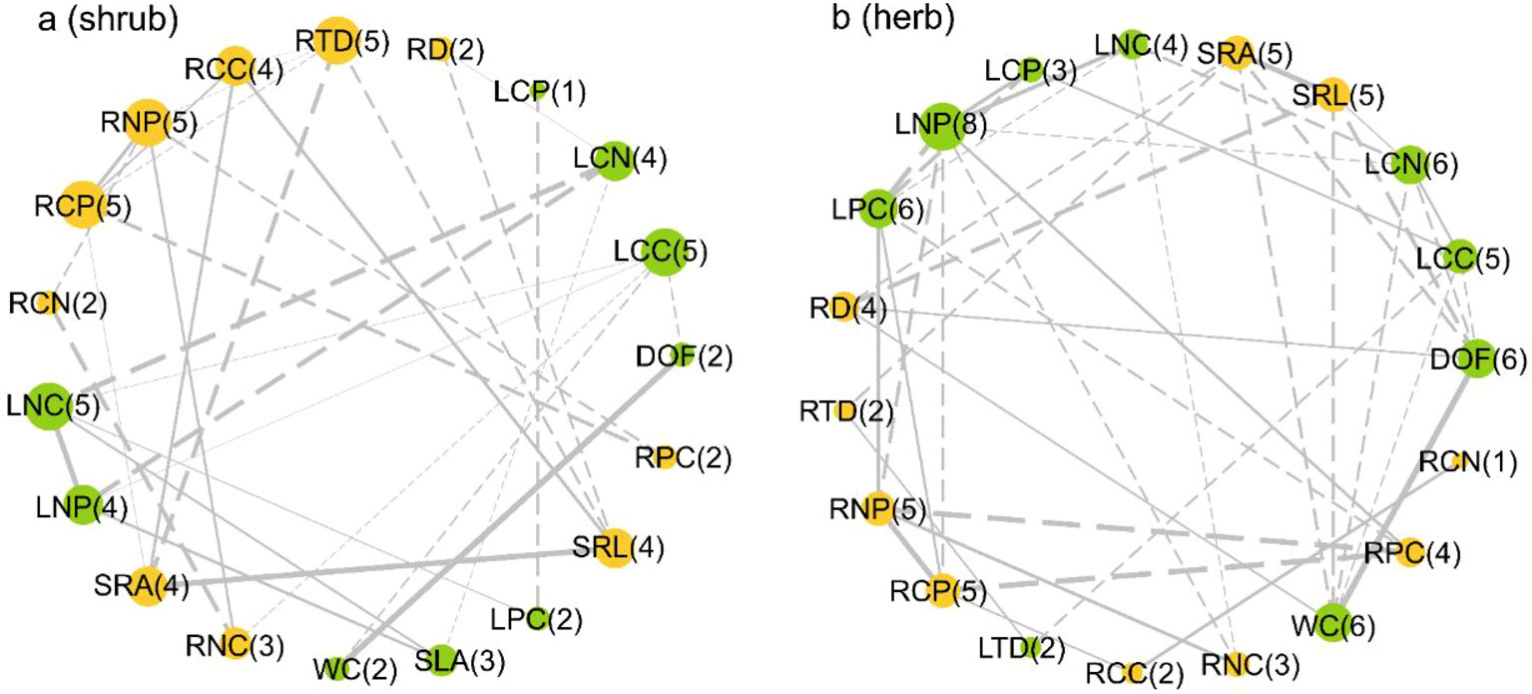
**(A)** The trait correlation network of shrub; **(B)** The trait correlation network of herb. Green nodes symbolize leaf functional traits, while orange nodes denote root functional traits. The size of a node reflects the extent of its connectedness. The number in parentheses following a trait’s abbreviation indicates the total number of connections that trait has with other traits (numbers in the figure indicate only the number of links between traits). Solid and dotted line represent positive and negative correlations (*p* < 0.05), respectively. DOF, Degree of fleshiness; WC, Leaf water content; SLA, Specific leaf area; LTD, Leaf tissue density; LCC, Leaf carbon content; LNC, Leaf nitrogen content; LPC, Leaf phosphorus content; LCN, Leaf carbon and nitrogen ratio; LNP, Leaf nitrogen and phosphorus ratio; LCP, Leaf carbon and phosphorus ratio; RD, Average root diameter; SRL, Specific root length; SRA, Specific root area; RTD, Root tissue density; RCC, Root carbon content; RNC, Root nitrogen content; RPC, Root phosphorus content; RCN, Root carbon and nitrogen ratio; RNP, Root nitrogen and phosphorus ratio; RCP, Root carbon and phosphorus ratio.

The results of trait network analysis showed that similar leaf and root traits defined in shrub species were completely decoupled (**Figure 2A**; *P* > 0.05); In herbaceous species, conceptually similar LTD was positively correlated with RTD, RNC was negatively correlated with LNC, RPC was negatively correlated with LPC, and RNP was negatively correlated with LNP (**Figure 2B**; *P* < 0.05). Herbaceous and shrub species had different hub traits (**Figure 2**). Among shrub species, RTD had the highest connectivity(largest node, highest total number of connections), followed by SRA and SRL, and the connectivity of root and leaf chemical traits was generally higher (**Figure 2A**). Among herbaceous species, LNP had the highest connectivity(largest node, highest total number of connections), followed by DOF, WC, SRA and SRL. Similar to shrub species, the connectivity of root and leaf chemical traits of herbaceous species was generally higher (**Figure 2B**).

### 3.2 Comparison of coefficient of variation among species for leaf and fine root traits

**Figure 3:**
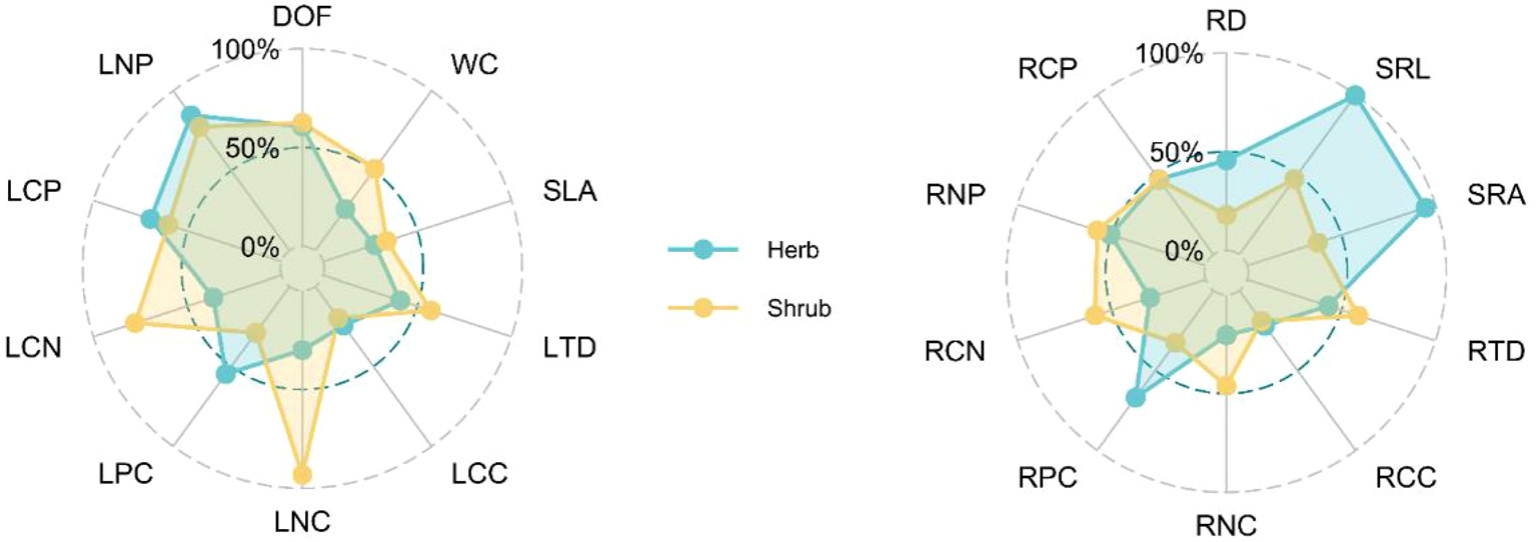
Interspecific variation coefficients of leaf and root functional traits.

The interspecific variation of root traits of shrubs was generally lower than that of leaf traits, and the variation of leaf traits of herbs was generally lower than that of root traits (**Figure 3**). In terms of leaf traits, the interspecific variation of shrubs was higher than that of herbs, and the LNC of shrubs had the highest interspecific variation. In terms of root traits, the variation of root traits of herbs was generally higher than that of shrubs, and the interspecific variation of SRL and SRA was the highest.

### 3.3 Principal component analysis of leaf, fine root and whole plant traits

**Figure 4:**
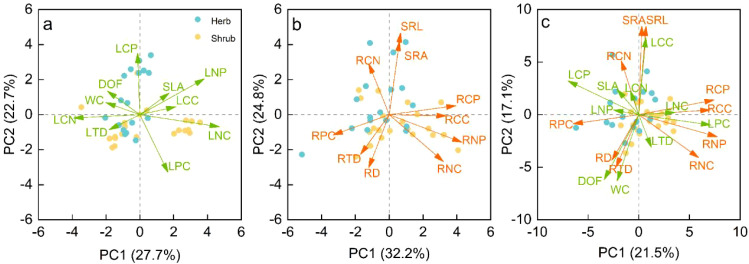
**(A)** Principal components analysis (PCA) of the leaf traits; **(B)** PCA of fine root traits; and **(C)** PCA of whole-plant traits. Blue nodes symbolize leaf functional traits, while orange nodes denote root functional traits. DOF, Degree of fleshiness; WC, Leaf water content; SLA, Specific leaf area; LTD, Leaf tissue density; LCC, Leaf carbon content; LNC, Leaf nitrogen content; LPC, Leaf phosphorus content; LCN, Leaf carbon and nitrogen ratio; LNP, Leaf nitrogen and phosphorus ratio; LCP, Leaf carbon and phosphorus ratio; RD, Average root diameter; SRL, Specific root length; SRA, Specific root area; RTD, Root tissue density; RCC, Root carbon content; RNC, Root nitrogen content; RPC, Root phosphorus content; RCN, Root carbon and nitrogen ratio; RNP, Root nitrogen and phosphorus ratio; RCP, Root carbon and phosphorus ratio.

Leaf, root, and whole plant principal component analysis showed that the PC1 axis explained 27.7%, 32.2% and 21.5% of the trait variation, and the PC2 axis explained 22.7%, 24.8% and 17.1% of the variation, and the cumulative contribution rate of the two variances was 50.4%, 57% and 38.6%, respectively (**Figures 4A–C**). In leaf traits, except for LCP and LPC, the contribution of other traits to the leaf PC1 axis was higher than that of the leaf PC2 axis (**Supplementary Table S2**). Among them, the traits LNC and SLA that had been defined as related to resource acquisition were mainly loaded positively along the leaf PC1 axis, while the conservative traits WC, DOF, and LTD had negative loads along the leaf PC1 axis (**Supplementary Table S2**). In root traits, root chemical traits contributed more to the PC1 axis (except RCN), and root morphological traits contributed more to the PC2 axis (**Supplementary Table S2**). RNC was loaded positively along the PC1 axis, and RTD was loaded negatively. SRL and SRA were loaded positively along the root PC2 axis, while RD was loaded negatively (**Supplementary Table S2**). In whole traits, RNC, LNC and LPC were loaded along the positive direction of whole-plant PC1, while LCP, RTD and RPC were loaded along the negative direction of whole-plant PC1. LCC, SRA and SRL were loaded along the positive direction of whole-plant PC2, while DOF, WC, RD and RTD were loaded along the negative direction of whole-plant PC2 (**Figure 4C**).

**Figure 5:**
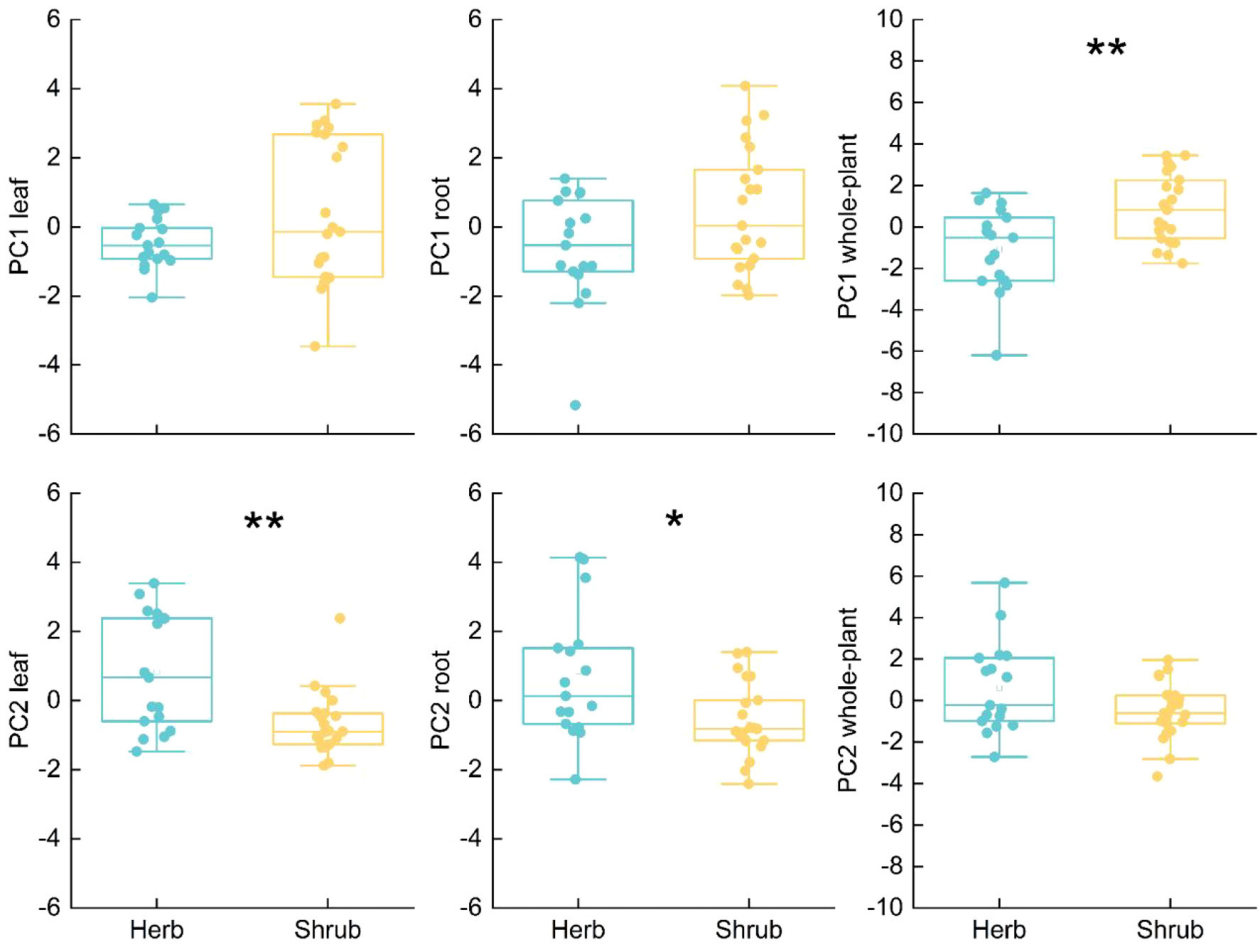
Results of independent sample t-test for elucidating the differences between herbs and shrubs species along PC1 and PC2. *P < 0.05, **P < 0.01.

There was no significant difference in leaf and root PC1 axis scores between herbaceous and shrub species (**Figure 5**). However, along the leaf PC2 axis, shrub species were distributed on the side with high LPC content, while herb species were distributed on the side with low LPC content (**Figure 4A**). However, along the root PC2 axis, herbaceous species were distributed on the side of high SRL and SRA, while shrub species were distributed on the side of high RD (**Figure 4A**). Therefore, a two-dimensional leaf economic spectrum and root economic spectrum could be defined. There were significant differences in the whole-PC1 axis scores between shrub and herb species (*P* < 0.01). Shrub species were distributed on the right side of the Whole-PC1 axis, and herb species were distributed on the left side of the PC1 axis (**Figure 4C**).

### 3.4 The relationship between plant economic spectrums

**Figure 6:**
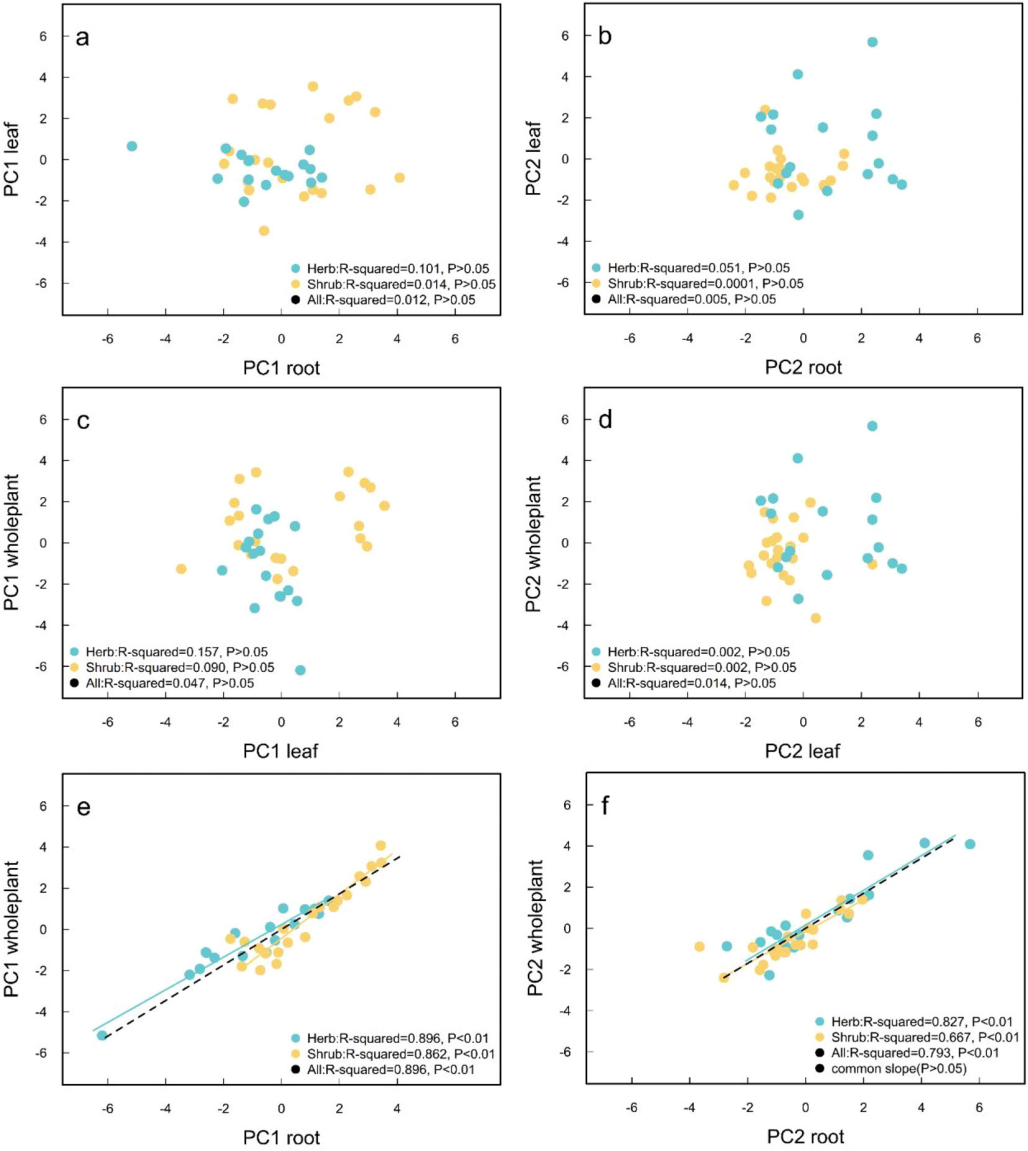
**(A)** Regression relationship between root PC1 and leaf PC1; **(B)** Regression relationship between root PC2 and leaf PC2; **(C)** Regression relationship between leaf PC1 and whole-plant PC1; **(D)** Regression relationship between leaf PC2 and whole-plant PC2; **(E)** Regression relationship between root PC1 and whole-plant PC1; **(F)** Regression relationship between leaf PC2 and whole-plant PC2. The yellow dots and lines represent shrub species. The blue dots and lines represent herb species. Black dots and lines represent all types. When *P* ≥ 0.05, the regression line was not drawn.

The SMA regression showed that leaf PC1 had no significant correlation with fine root PC1 and whole-plant PC1 (*P* > 0.05) (**Figures 6A, C**). There was no significant correlation between leaf PC2 and fine root PC2 and whole-plant PC2 (*P* > 0.05) (**Figures 6B, D**). For all species studied, fine root PC1 was significantly correlated with whole-plant PC1 (*P* < 0.01) (**Figure 6E**), and fine root PC2 was significantly correlated with whole-plant PC2 (*P* < 0.01) (**Figure 6F**). The fine root PC1 of herbs and shrubs was significantly correlated with the whole-plant PC1 (*P* < 0.05), and there was no common slope between herbs and shrubs (*P* < 0.05). Fine root PC2 was significantly correlated with whole-plant PC2. There was a common slope between herb species and shrub species (*P* > 0.05), but there was no common intercept (*P* < 0.05) (**Figures 6A, C**).

## 4 Discussion

### 4.1 Coordination of traits between and within organs

Our results show that the leaf and root traits that are similar in the definition of shrub species are completely decoupled, e.g., there were no correlation between leaf tissue density (LTD) and root tissue density (RTD), leaf nitrogen content (LNC) and root nitrogen content (RNC), leaf phosphorus content (LPC) and root phosphorus content (RPC), and leaf nitrogen and phosphorus ratio (LNP) and root nitrogen and phosphorus ratio (RNP) (**Figure 2A**), while some conceptually similar and key functional traits of herbaceous plants are highly coordinated, such as positive correlation between LTD and RTD, negative correlation between RNC and LNC, negative correlation between RPC and LPC, and negative correlation between LPC and LNP. (**Figure 2B**). This may be due to the higher mycorrhizal colonization rate of woody plants compared to herbs, resulting in the fact that similar traits are not necessarily the same in function (Luo et al., 2024). Specifically, compared with the softer and finer root tissues of herbaceous plants, the thicker root tissues of woody plants are more conducive to the colonization of mycorrhizal fungi (Ma et al., 2018), which makes woody plants with lower specific root length can also obtain nutrients by colonization of higher mycorrhizal fungi (Bergmann et al., 2020). However, herb roots are not conducive to mycorrhizal fungi colonization (Ma et al., 2018), and rely more on the larger specific root lengths for obtain nutrients. The decoupling characteristics between organs of shrub plants indicate that leaf and root traits evolve independently, allowing shrubs to have more trait combinations to cope with desert environments and promote local coexistence of species (Reich, 2014; Weemstra et al., 2021; Yu et al., 2022; Luo et al., 2024). On the contrary, the N content in roots and leaves of herbaceous plants was significantly negatively correlated with P content, while the tissue density was significantly positively correlated (**Figure 2B**), indicating that there was an inherent physiological and life history trade-off between leaves and roots in desert herbs (Laughlin et al., 2010; Hu et al., 2019). That is to say, there is a basic trade-off between nutrient acquisition efficiency and photosynthetic activity of herbaceous plants in this area, which is conducive to plants to adopt optimal resource acquisition strategies to alleviate the limitations of extreme environments.

The different degree of interdependence between traits has a profound impact on the coordination of different organs and tissue systems in response to environmental changes (Freschet et al., 2015a; Li et al., 2022). Therefore, there is a complex interaction network between different traits of different organs and tissue systems (Proulx et al., 2005). In the network, changes in some hub traits may lead to changes in the trade-offs between traits (Li et al., 2024). The results of our network analysis showed that the connectivity of chemical traits was generally higher than that of morphological traits in both herbaceous and shrub species (**Figures 2A, B**). This can be attributed to the important role of nitrogen and phosphorus in plant metabolism, function, growth and reproduction (Kerkhoff et al., 2006; Li et al., 2010; Crous et al., 2017). Specifically, plants absorb nitrogen and phosphorus from the soil through roots and transport them to other organs of plants to meet the functional needs of individual plants (Reich, 2014; Chen et al., 2020). Therefore, it is not surprising that chemical traits have higher connectivity. Consistent with our hypothesis (i), both shrubs and herbs showed higher connectivity in traits related to nutrient acquisition and storage [such as specific root length (SRL), specific root area (SRA), degree of fleshiness (DOF) and leaf water content (WC)] (**Figures 2A, B**). This may be because the species we collected are mostly distributed in desert habitats, where water is an important limiting factor for plant growth (Wang et al., 2024), and water is the basis for the transport and function of various chemicals. Therefore, it is expected that desert plants have a high degree of connectivity with traits related to water acquisition and storage.

### 4.2 Interspecific variation and resource economics spectrum strategies of root and leaf traits

The variation of interspecific functional traits not only helps us to understand the evolutionary adaptation strategies of different species (Li et al., 2024), but also reflects the influence of environmental filtering and competition on plant functional traits at the regional scale (Luo et al., 2024). Consistent with our hypothesis (ii), our results show that the interspecific variation of root traits is higher than that of leaf traits in shrubs, while the opposite is true in herbaceous plants (**Figure 2**). This indicates that under the combined action of environmental filtering and interspecific competition, the strategy of the aboveground and underground parts of herbs and shrubs occupying niches is decoupled. Specifically, shrubs rely on leaf variation to occupy different niches, while herbs rely more on root variation to achieve this goal. In addition, our study also found that the leaf nitrogen content of desert shrubs and the specific root length and specific root surface area of herbs have very high interspecific variation, indicating that the leaf metabolic activity of shrubs and the root absorption efficiency of herbs are strongly affected by interspecific competition, which makes different species show divergent adaptation (Wang et al., 2024). This divergent adaptation is beneficial for desert plants to adopt different life history strategies to reduce biological competition and resource constraints when dealing with highly resource-constrained desert environments (Yu et al., 2022).

Life form can regulate the variation of leaf and root traits at the same time, so that herbaceous and woody plants have different strategies of resource economic spectrum (Freschet et al., 2017; Kong et al., 2019). The results based on principal component analysis showed that the root, leaf and whole-plant economic spectra of the species we studied were all two-dimensional (**Figures 3A, B**). Herb and shrub species diverged along the PC2 axis of RES and LES. Herb species had higher SRL, SRA and lower LNP, while shrub species had higher RD and LNP. This indicates that in desert environments, shrubs rely mainly on leaf metabolism (Adler et al., 2014) and mycorrhizal colonization (Bergmann et al., 2020) to compete for nutrients, while herbs rely more on the high resource acquisition efficiency of underground roots (Roumet et al., 2016). Interestingly, for the plant strategy, herbaceous species were distributed on the higher RTD and tissue C/N ratio side of whole plant economics spectrum (WPES), while shrub species were distributed on the higher tissue N and P concentration side. This result is contrary to the analysis results based on global data that woody plants have higher tissue density (Freschet et al., 2017; Jiang et al., 2024). The result of this difference may be due to the greater ecological challenges faced by herbaceous species in the desert environment. First, shrubs can store more water by using coarse canopy and loose xylem liking porous sponges (McElrone et al., 2004). Secondly, shrubs can concentrate nutrients around themselves by changing soil structure and humidity, so as to ensure their normal physiological metabolism. However, due to the strong competition of shrubs for water and nutrients, herbaceous plants in the same environment can only adopt a relatively conservative resource acquisition strategy. In addition, shrub roots usually have higher cortical thickness, which is of great significance in resisting underground animal grazing, while herbaceous plants seem to rely only on tougher roots.

### 4.3 Resource economic spectrum coordination model from organ to individual

Although different traits are in a constant state of change due to species differences and environmental changes, the trade-offs and synergies between traits are relatively stable ecological strategies formed by adaptive evolution (Eviner and Iii, 2003). Consistent with our hypothesis (iii), Our study shows that the LES trade-off axis and the RES trade-off axis operate independently for both desert herbs and shrub species. This is inconsistent with the functional similarity hypothesis and deviates from the results of some studies (Poorter et al., 2014; Zhao et al., 2017; Li et al., 2022). However, the present results are consistent with some research results, that is, different organizations are decoupled from each other and adapt to environmental changes independently (Baraloto et al., 2010; Isaac et al., 2017; Kurze et al., 2021). On the one hand, these differences may arise from the distinct structural and functional characteristics of the organs. Desert plants possess thicker fleshy tissues, cuticles, and waxy layers, which enhance water storage and minimize water loss. In contrast, fine roots typically exhibit a slender morphology and a high surface area-to-volume ratio, facilitating extensive soil penetration and efficient water and nutrient absorption (Ekwealor et al., 2020). On the other hand, these differences may reflect the divergent survival strategies of different organs in heterogeneous environments. Leaves primarily acquire resources from the atmosphere, while fine roots are responsible for extracting soil resources. This functional independence allows each organ to adapt flexibly to spatial variations in resource availability, thereby reducing the overall risk to plant survival and providing a competitive advantage in resource-poor habitats (Kirschner et al., 2021; Tariq et al., 2024). In addition, our study also shows that LES and WPES are also decoupled, which is consistent with the results in tropical woody plants (Baraloto et al., 2010). The reason may be that previous studies were mostly based on trees rather than shrubs and herbs. They mostly grow in areas with abundant precipitation, soil nutrients, and high biodiversity, leading to more intense interspecific competition. Therefore, more robust leaf and root coordination is needed to improve their ability to compete for resources (Li et al., 2022).

Fine root PC1 and whole-plant PC1 reflect the trade-off between plant metabolic activity and stress resistance. Our study found that fine root PC1 of herbs and shrubs was significantly positively correlated with whole-plant PC1 and the slope of shrubs was greater than that of herbs. This may be because desert shrubs have deep roots (Kirschner et al., 2021), coupled with their well-developed fine roots that exhibit complex growth regulation mechanisms, robust absorptive capacity, and high-stress resistance. Even minor variations in these traits can significantly impact the entire plant (Danin, 1991). Additionally, the resource-scarce nature of desert habitats means that fine root traits are critical for resource acquisition. Desert shrubs usually occupy large ecological niches, and their considerable height and crown width. Fine root traits are intricately linked to the absorption and transport of materials and energy throughout the plant, thereby establishing a strong correlation between fine root characteristics and the overall growth and development of the plant (Comas and Eissenstat, 2009). In conclusion, whether plants improve metabolic activity or stress resistance depends on the ability of developed roots to acquire and transport groundwater. Therefore, the coordination between shrub fine roots and plant economic spectrum is stronger. In contrast, the roots of desert herbaceous plants are mostly distributed in the shallow soil layer (<30cm) (Jackson et al., 1996). These plants exhibit short growth cycles, simple regulatory mechanisms, and narrow ecological niches. Their above-ground parts are lesser, and their requirements for access to soil resources are relatively straightforward, leading to a weak integration between fine roots and the overall plant structure (Lambers and Oliveira, 2019). Although the metabolic activity and stress resistance selection of plant individual are also dependent on fine roots to a certain extent, the instability of water and nutrient supply in fine roots reduces the dependence of plants on them. Fine root PC2 and whole-plant PC2 reflect the trade-off between water absorption and water retention of plants. Our study found that fine root PC1 of herbs and shrubs was significantly positively correlated with whole-plant PC1 and the slope was the same, but the intercept was different. It shows that shrubs and herbaceous species respond to changes in growth characteristics through the separation of intercepts in the regression relationship. On the one hand, similar slopes indicate that shrub and herb species have consistent resource acquisition and preservation strategies at the organ and plant levels (Freschet et al., 2010). On the other hand, differences in intercepts indicate that shrubs and herbs have different economic spectrum strategies (Yu et al., 2022).

## 5 Conclusion

This study reveals the variation and coordination patterns of leaf and root traits in desert plants. The results showed that the independent variation of leaf and fine root traits of desert plants along the two axes did not depend on life form, and the traits related to water acquisition and preservation were important hub traits. Multidimensional variation of root and leaf traits of desert shrubs and herbs is more complicated, and they cannot be simply divided into acquired and preserved species because they all adopt trait syndromes that are beneficial to themselves.

## Data Availability

The original contributions presented in the study are included in the article/**Supplementary Material**. Further inquiries can be directed to the corresponding author.
