# Supplementary material for: Discrepancy in coordination and variation of root and leaf traits among herbaceous and shrub species in the desert, China
Source: Front Plant Sci. 2024 Nov 12;15:1485542. doi: 10.3389/fpls.2024.1485542 (PMC11589817; doi:10.3389/fpls.2024.1485542)
Supplement: Supplementary file 1 [file Table1.docx]

**Appendix A. Supplementary data**

Table S1 Correlation coefficients among the measured leaf and fine root functional traits

| Traits | RD | SRL | SRA | RTD | RCC | RNC | RPC | RNP | RCP | RCN |
| --- | --- | --- | --- | --- | --- | --- | --- | --- | --- | --- |
| DOF | 0.24 | -0.48** | -0.50** | 0.30 | -0.25 | -0.03 | -0.03 | -0.08 | -0.11 | -0.07 |
| WC | 0.22 | -0.46** | -0.49** | 0.28 | -0.26 | -0.05 | -0.02 | -0.10 | -0.12 | -0.06 |
| SLA | -0.24 | 0.20 | 0.10 | 0.00 | -0.27 | -0.28 | -0.17 | -0.15 | -0.01 | 0.12 |
| LTD | **-0.33*** | 0.12 | 0.05 | 0.16 | 0.14 | 0.14 | 0.16 | 0.08 | -0.05 | -0.09 |
| LCC | -0.10 | **0.32*** | 0.35* | -0.24 | 0.07 | -0.30 | -0.26 | -0.01 | 0.21 | 0.32 |
| LNC | -0.09 | -0.11 | -0.15 | 0.21 | 0.14 | -0.07 | 0.09 | -0.13 | -0.06 | 0.01 |
| LPC | -0.06 | 0.10 | 0.14 | -0.15 | **0.47**** | **0.42**** | -0.25 | **0.38*** | **0.36*** | -0.12 |
| LCN | 0.10 | 0.11 | 0.14 | -0.15 | -0.19 | -0.08 | -0.09 | 0.02 | 0.04 | 0.13 |
| LCP | 0.05 | 0.04 | 0.03 | 0.01 | -0.30 | **-0.49**** | 0.10 | **-0.33*** | -0.20 | 0.25 |
| LNP | 0.00 | -0.11 | -0.13 | 0.15 | 0.01 | -0.22 | 0.22 | -0.28 | -0.21 | 0.04 |


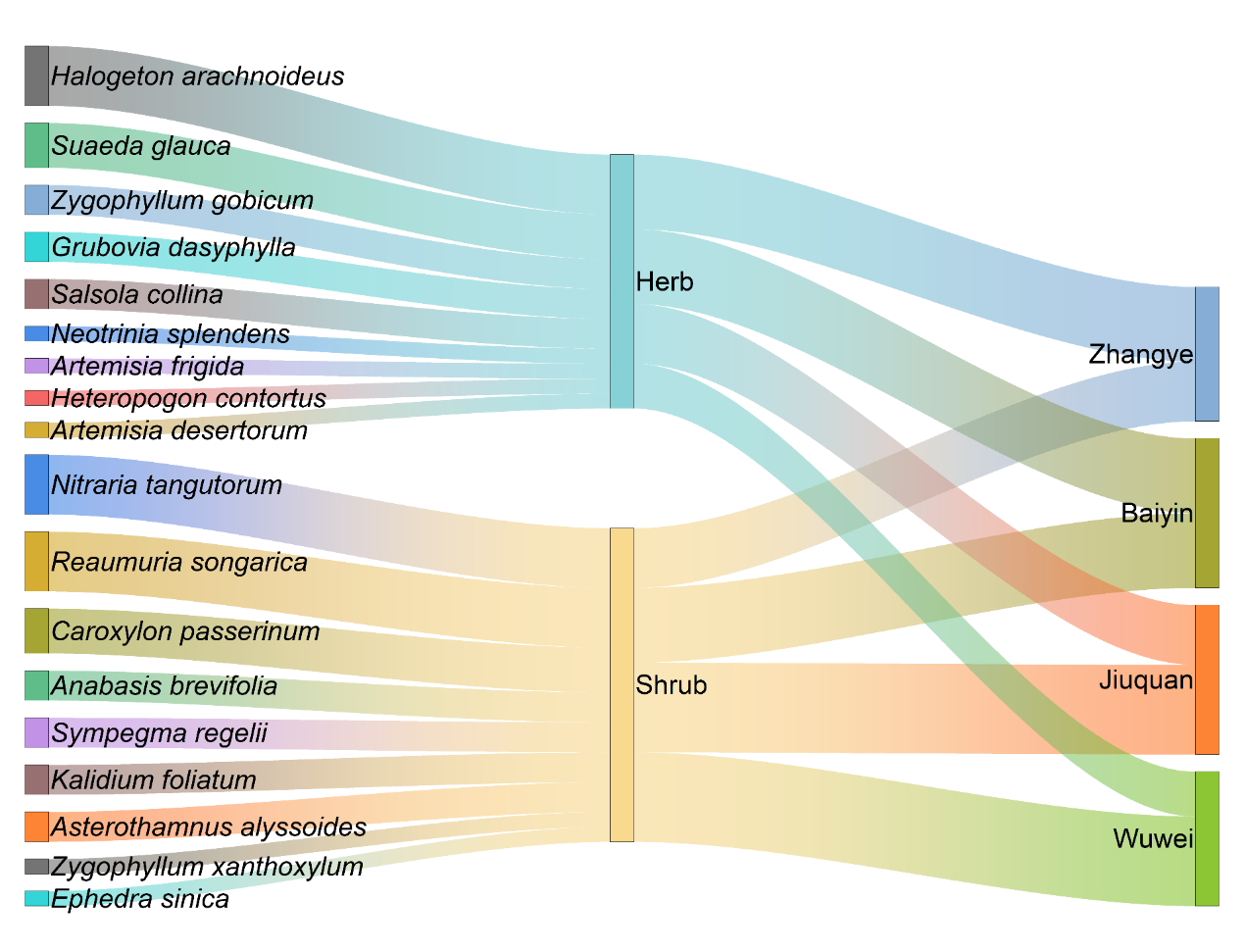


Figure S1 Collection information of species from desert habitats in Gansu province, China.

Table S2

|  | Leaf | |  | Root | |  | Whole-plant | |
| --- | --- | --- | --- | --- | --- | --- | --- | --- |
|  | PC1 | PC2 |  | PC1 | PC2 |  | PC1 | PC2 |
| DOF | -0.32* | 0.39* |  |  |  |  | -0.32* | -0.62*** |
| WC | -0.32 | 0.40* |  |  |  |  | -0.34* | -0.60*** |
| SLA | 0.52*** | 0.48** |  |  |  |  | -0.16 | 0.30 |
| LTD | -0.34* | -0.31 |  |  |  |  | 0.11 | -0.09 |
| LCC | 0.51** | 0.15 |  |  |  |  | 0.08 | 0.70*** |
| LNC | 0.88*** | 0.33* |  |  |  |  | 0.00 | 0.04 |
| LPC | 0.25 | -0.71*** |  |  |  |  | 0.72*** | 0.02 |
| LCN | -0.69*** | -0.16 |  |  |  |  | -0.12 | 0.19 |
| LCP | 0.12 | 0.73*** |  |  |  |  | -0.57*** | 0.32* |
| LNP | 0.80*** | 0.59*** |  |  |  |  | -0.26 | 0.06 |
| RD |  |  |  | -0.01 | -0.57*** |  | -0.05 | -0.51*** |
| SRL |  |  |  | 0.30 | 0.73*** |  | 0.30 | 0.73*** |
| SRA |  |  |  | 0.45** | 0.66*** |  | 0.41* | 0.68*** |
| RTD |  |  |  | -0.46** | -0.28 |  | -0.41* | -0.35* |
| RCC |  |  |  | 0.66*** | -0.05 |  | 0.71*** | 0.09 |
| RNC |  |  |  | 0.60*** | -0.64*** |  | 0.60*** | -0.49** |
| RPC |  |  |  | -0.70*** | -0.19 |  | -0.57*** | -0.24 |
| RCN |  |  |  | -0.24 | 0.71*** |  | -0.21 | 0.56*** |
| RNP |  |  |  | 0.91*** | -0.28 |  | 0.81*** | -0.14 |
| RCP |  |  |  | 0.87*** | 0.10 |  | 0.79*** | 0.21 |
